# A global record of annual terrestrial Human Footprint dataset from 2000 to 2018

**DOI:** 10.1038/s41597-022-01284-8

**Published:** 2022-04-19

**Authors:** Haowei Mu, Xuecao Li, Yanan Wen, Jianxi Huang, Peijun Du, Wei Su, Shuangxi Miao, Mengqing Geng

**Affiliations:** 1 College of Land Science and Technology, China Agricultural University, Beijing, 100083 China; 2 Key Laboratory of Remote Sensing for Agri-Hazards, Ministry of Agriculture and Rural Affairs, Beijing, 100083 China; 3 School of Geography and Ocean Science, Nanjing University, Nanjing, Jiangsu 221100 China

**Keywords:** Environmental impact, Conservation biology, Population dynamics

## Abstract

Human Footprint, the pressure imposed on the eco-environment by changing ecological processes and natural landscapes, is raising worldwide concerns on biodiversity and ecological conservation. Due to the lack of spatiotemporally consistent datasets of Human Footprint over a long temporal span, many relevant studies on this topic have been limited. Here, we mapped the annual dynamics of the global Human Footprint from 2000 to 2018 using eight variables that reflect different aspects of human pressures. The accuracy assessment revealed a good agreement between our mapped results and the previously developed datasets in different years. We found more than two million km^2^ of wilderness (i.e., regions with Human Footprint values below one) were lost over the past two decades. The biome dominated by mangroves experienced the most significant loss (i.e., above 5%) of wilderness, likely attributed to intensified human activities in coastal areas. The derived annual and spatiotemporally consistent global Human Footprint can be a fundamental dataset for many relevant studies about human activities and natural resources.

## Abstract (summary)

**Table:** 

Measurement(s)	human footprint
Technology Type(s)	remote sensing
Factor Type(s)	Built Environment • Population Density • Nighttime lights • Croplands • Pastures • Roads • Railways • Navigable waterways

## Background & Summary

The intensified human activities are influencing the ecological processes and anthropogenic biomes^1^, causing distinct changes in species distributions and habitats^2^. Globally, biodiversity is declining at an alarming rate due to the increased risk of species extinction caused by human activities^3,4^. Most studies about human activities investigate the conversion of land cover and land use alone^4,5^, which are inadequate to capture diverse pressures from human activities. Meanwhile, some studies considering the single pressure (e.g., nighttime light^6^ or population density^7^) are limited in exploring the synthesized effect of multiple human activities^8,9^. Presently, many ecosystems suffer various ecological and environmental pressures beyond their tolerances for recovery^10^. Consequently, mapping spatiotemporally consistent datasets of Human Footprint is urgently required in practical applicatioins^11^.

Studies of mapping global human pressures have been conducted to understand the influence of humans on habitat and biodiversity. The first temporally comparable global Human Footprint maps were developed by Venter *et al*.^12,13^ with two phases (i.e., 1993 and 2009). These maps have been extensively used in studies about biodiversity^12^, ecological landscape^14,15^, and climate change^10,16^. New definitions were derived using the conventional approach of mapping Human Footprint^13,17^, including the wilderness (Human Footprint < 1), the intact areas (Human Footprint < 4), and the highly modified (Human Footprint ≥ 4) regions^18,19^. The Human Footprint data can greatly extend their applications under different scenarios^20–24^. For example, Watson *et al*.^25^ investigated the relationship between Human Footprint and native forests. They found the integrity of intact forest ecosystems is crucial to maintaining biodiversity. Marco *et al*.^20^ found the wilderness derived from Human Footprint can significantly reduce the rate of species loss than that in non-wilderness areas.

Previous studies about Human Footprint mapping mainly focus on the spatial heterogeneity of the derived results, with little consideration of the temporal dynamics of human activities^13,18,23^. This significantly limited the wide application of Human Footprint maps in practical applications. Humans have considerably impacted the natural ecosystems in the Anthropocene over the past decades^26^. However, due to the rapid urbanization and population increase, mapping Human Footprint with relatively coarse temporal resolution (e.g., five years or decade) is inadequate, particularly when facing rapidly changing environments (e.g., urbanization). Hence, a consistent record of Human Footprint across space and time is of great importance to evaluate human-induced changes and promote sustainable development.

In this study, we developed annual records of the global Human Footprint dataset from 2000 to 2018, using eight variables (i.e., built environment, population density, nighttime lights, cropland, pasture, roads, railways, and navigable waterways). First, we adopted a standard mapping framework to characterize the level of Human Footprint with consistent definitions across space and time. Then, we evaluated our results using validation samples collected from the visual interpretation and compared our derived maps with other studies across different years. Finally, we investigated the dynamics of wilderness and highly modified areas across different terrestrial biomes^27^.

## Methods

**Fig. 1:**
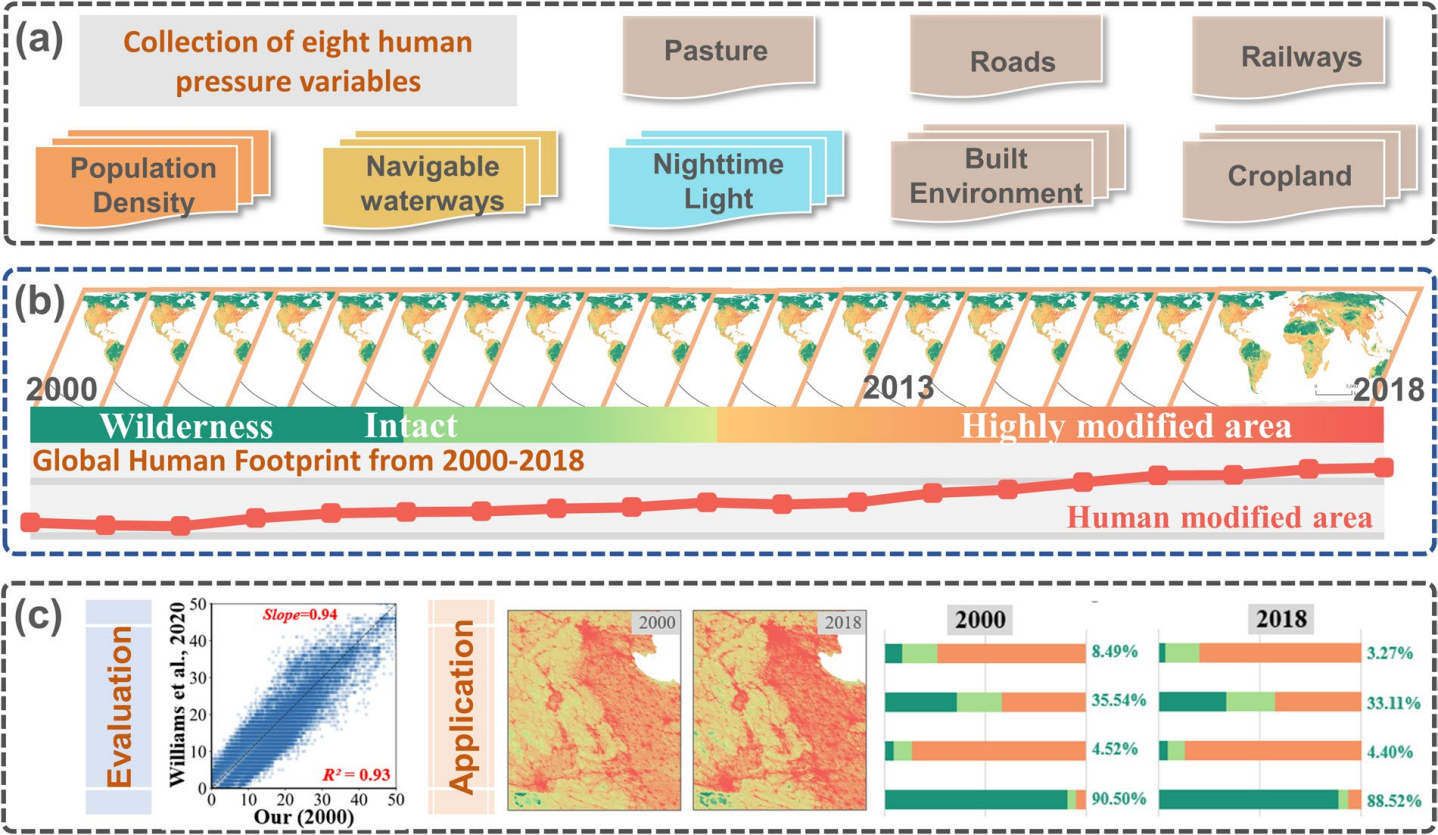
The proposed framework in this study by collecting eight human pressure variables from multiple sources (**a**), generating annual global Human Footprint datasets (**b**), and the evaluation and application of derived results (**c**).

We generated the annual records of the global Human Footprint from 2000 to 2018 using eight variables that characterize the human pressures (Fig. 1). The proposed framework includes three components. First, we collected and processed eight variables that reflect human pressures from different aspects, such as land transformation, population density, human access, and infrastructures (Fig. 1a). Then, we generated the time series data of the annual global Human Footprint using consistent definitions and mapping framework (Fig. 1b). Finally, we evaluated the derived results through comparison with the validation samples and previous studies to explore the dynamics of Human Footprint across different global terrestrial biomes^27^ (Fig. 1c).

### Human pressure variables

We employed eight pressure variables that reflect different aspects in our study, including built environments, population density, nighttime light, croplands, pasture lands, roadways, railways, and navigable waterways. Unlike previous studies that only use limited epochs of pressure variables^18,23^, we improved the temporal consistency of some crucial pressure variables, such as the annual maps of global artificial impervious area (GAIA)^5^ and the global harmonized nighttime light dataset^28^. These pressure variables were preprocessed to 1 km resolution with global coverage. We followed the classic method developed by Sanderson and Venter^12,13,17^ to generate Human Footprint datasets. Details of each pressure variable can be found in the following sections.

#### Built environments

The expansion of built environments is threatening the biodiversity in species-rich regions^29^. As the dominant change in the built environment, the process of urban sprawl can be quantitatively characterized by remotely sensed observations^5,30,31^. The expansion of impervious surface areas, commonly composited by artificial materials (e.g., roofs, paved surfaces, hardened grounds, and major road surfaces) in the built environment^32–35^, would fragment the natural habitats and disturb the richness of species^36,37^. Satellite images can detect the dynamics of impervious surface area and further support the mapping of the Human Footprint.

We adopted the GAIA data in this study to represent the built environment. Given that the spatial resolution of GAIA is 30 m, we calculated the urban fraction within the 1 km grid and regarded those pixels with percentages above 20% as urban^38^. Compared to previous studies that generate the built environment through nighttime light (NTL)^13,18^, the GAIA data are advanced regarding the temporal consistency across different years and the improved performance in delineating the urban extent. For example, there are some limitations using the NTL data as the variable for Human Footprint mapping, such as the overflow and saturation effects of NTL data in/around the city and the absence of inter-calibration of NTL time series data among the sensors and satellites^39–42^.

#### Population density

With the increase of population, human-induced environmental changes are likely to threaten biodiversity and degrade the environment of habitats^43,44^. Hence, in this study, we used the population density data collected from the WorldPop program^45,46^, which provides temporally consistent maps of the population with a medium resolution of 100 m. We aggregated the population density data to 1 km as well.

#### Nighttime lights

Nighttime light provides a unique aspect to detect human activities from satellites, showing great potential in measuring the human pressures on natural ecosystems^47,48^. Previous studies of mapping Human Footprint using NTL data are limited due to the temporal inconsistency of NTL observations from the raw Defense Meteorological Satellite Program (DMSP) data (1992–2013), as well as the difference of NTL data between the DMSP and the Visible Infrared Imaging Radiometer Suite (VIIRS) (2012-now)^18,28,40^. These limitations have been significantly improved with the advent of newly developed NTL datasets^28^. In this study, we employed the harmonized NTL dataset at the global scale, which integrated the inter-calibrated NTL observations from the DMSP and the simulated DMSP-like NTL observations from the VIIRS data with a high degree of temporally consistency^40^.

#### Crop and pasture lands

In addition to urban lands, the expansion of cropland and pasture lands is another source of human activities that may cause habitat loss and the degradation of biodiversity^49,50^. We used the annual crop maps derived from the European Space Agency (ESA) Climate Change Initiative (CCI) Landcover dataset (http://maps.elie.ucl.ac.be/CCI/viewer/)^51^. Also, we employed the widely used pasture map developed by Ramankutty *et al*.^52^, which combined agriculture census data and satellite-derived land cover and has been extensively used in Human Footprint mapping^52,53^. It is worth noting that the pasture map is consistent across years without annual change information.

#### Roads and railways

Roads are links between natural and human environments and are highly related to human activities^54,55^. Here, we obtained the global roads by combining records in the Open Street Maps (OSM) (https://planet.osm.org) and the Global Roads Open Access Dataset (gROADS)^56^. The gROADS contains the most available road data in each country, whereas the OSM is a volunteer-driven, open-source global mapping project that contains freely accessible detailed geographic information around the world. In this study, all trails and minor roads were excluded. Besides, the railways were collected from the National Geospatial-Intelligence Agency (NGA; https://gis-lab.info/qa/vmap0-eng.html).

#### Navigable waterways

Navigable waterways are other corridors that link the aquatic environment and human activities by ships and pollution^57,58^. We quantified the pressure indicated by navigable waterways following the approach of Human Footprint in Venter *et al*.^13^. The navigable waterways were determined by (1) the river depth is greater than 2 m, and (2) the distance to lit pixel is within 4 km. In this study, we mapped global navigable waterways by integrating the coasts and rivers from NGA and the HydroSHEDS (Hydrological data and maps based on SHuttle Elevation Derivatives at multiple Scales)^59^. We determined the annual navigable waterways by comparing the river networks with the annual NTL data from 2000 to 2018.

### Mapping of annual Human Footprint data

We mapped the annual dynamics of Human Footprint at the global scale using the standard framework developed by Venter and Williams *et al*.^13,18^. In the beginning, all these eight human pressure variables were preprocessed to 1 km. Then, different scores were assigned according to their contributions (Table 1). Given that variables used to characterize the built environments and cropland were derived from the high-resolution datasets, we assigned their scores according to their fractions within the pixel. For example, when the fraction of urban is greater than 20%, the built environment was assigned a score of 10. The population density was assigned with a pressure score of 10 for pixels with more than 1000 people in each 1 km grid, while for those pixels with densities less than 1000, their pressure scores were measured using the equation in Table 1. Besides, we measured the direct and indirect influence of traffic networks according to the distance of each pixel to nearby roads and railways. For pixels close to the roads and railways, we assigned the score of 8 as suggested in Venter *et al*.^13^; otherwise, we assigned their scores according to the distance decaying relationship in Table 1.

**Table 1:** The framework of mapping annual Human Footprint at the global scale as illustrated in Williams *et al*.^18^.

Pressure	Score	Details
Built environment	0,4,10	The pressure score for pixels with urban fractions above 20% was assigned as 10; otherwise, it was assigned as 4.
Population density	0–10	$$\left\{\begin{array}{l}10,\;population\left(P\right)\ge 1000\\ 3.333\times \log \left(P+1\right),0 < P < 1000\end{array}\right.$$
Night-time lights	0–10	Assigned from 0 to 10 according to intervals determined by ten equal quantiles
Croplands	0,4,7	The pressure score for pixels with crop fraction above 20% was assigned as 7; otherwise, it was assigned as 4.
Pasture	0–4	Fraction of pasture in each grid multiplied by 4
Roads	0–8	$$\left\{\begin{array}{l}8,distance\;\left(D\right)\le 0.5\\ 3.75\times \exp \left(-1\times \left(D-1\right)\right)+0.25,0.5 < D < 15\end{array}\right.$$
Railways	0,8	$$\left\{\begin{array}{l}0,distance\;\left(D\right) > 0.5\\ 8,D\le 0.5\end{array}\right.$$
Navigable waterways	0–4	$$\left\{\begin{array}{l}0,distance\;\left(D\right) > 15\\ 4\times \exp \left(-1\times D\right),D < 15\end{array}\right.$$

### Evaluation and application of the derived datasets

We evaluated the derived Human Footprint maps through comparison with previous studies^13,18,24^, and analyzed the temporal trends of the derived dataset in different terrestrial biomes^27^. We assessed our results using visually interpreted samples (in total: 3,460) from Venter^13^. The degree of Human Footprint in these samples was interpreted according to the shape, size, texture, and color of human-related features (e.g., built environment, cropland, and road) in high resolution satellite images. We also compared our mapped results with previously developed products in literature, such as maps in Venter *et al*.^13^, Williams *et al*.^18^, and Kennedy *et al*.^24^ at multiple phases. In addition, we also explored the dynamics of Human Footprint at the globe across different terrestrial biomes, including specific types such as wilderness (Human Footprint of < 1), intact areas (Human Footprint of < 4), and highly modified areas (Human Footprint of ≥ 4).

## Data Records

The annual records of the global Human Footprint from 2000 to 2018 can be accessed freely at the figshare repository (10.6084/m9.figshare.16571064)^60^. All Human Footprint were mapped using the Mollweide equal-area projection at 1 km resolution. The data collection contains one .zip file for each year, labelled hfpXXXX.zip. Each .zip file contains one GEOTIFF.

## Technical Validation

**Fig. 2:**
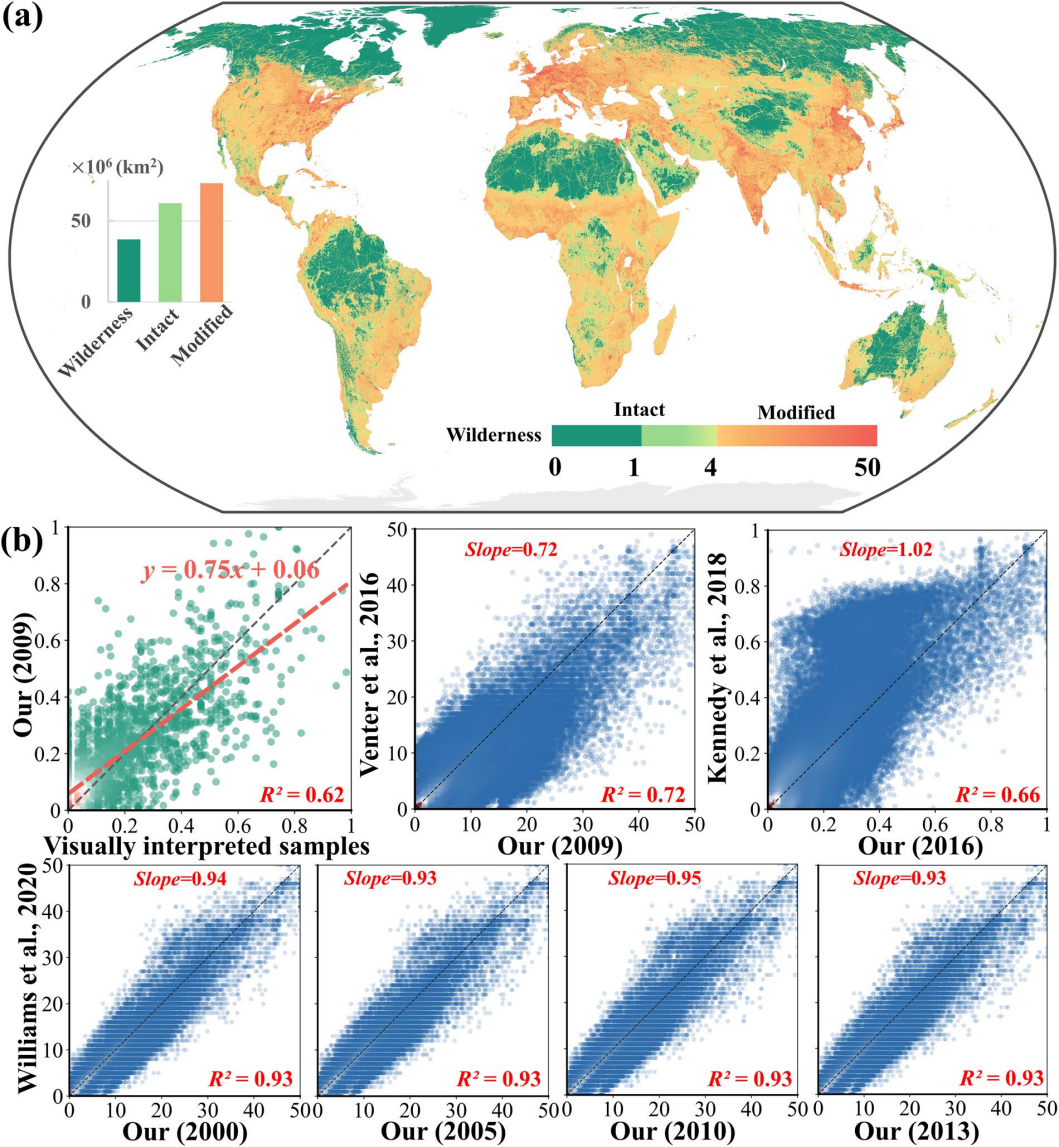
The derived Human Footprint map in 2009 (**a**) with evaluations using interpreted samples and other published products (**b**). We normalized our results for comparison because the ranges of visually interpreted samples and Kennedy’s result^24^ are 0-1, and visually interpreted samples from Venter *et al*.^13^.

The comparison between our Human Footprint datasets and other studies suggests a high degree of consistency. Globally, only about one-third of the global land (38.6 million km^2^) is wilderness (less than 1) in 2009, the year of the referred Human Footprint data in Venter *et al*.^13^, whereas areas of intact (less than 4) and highly modified areas (greater than 4) are 60.9 million km^2^ and 73.2 million km^2^, respectively (Fig. 2a). This result suggests our planet has been notably impacted by humans worldwide, showing similar results as the target of protecting half of the natural lands^61^. The validation using visually interpreted samples reveals an improved correlation with R^2^ of 0.62, higher than that in previously developed Human Footprint map (R^2^ is 0.50)^13^ (Fig. 2b). The improved correlation with interpreted samples is attributable to the improved human pressure variables adopted in this study. Meanwhile, our derived results show a high agreement with other studies regarding indicators of the slope and R^2^, in particular with results from William’s^18^ in different years (i.e., 2000, 2005, 2010, and 2013) and from Kennedy’s^24^ in 2016 (Fig. 2b). It is worth noting that the temporal span in our results is expanded with a high degree of temporal consistency compared with other studies. Thus, the derived results can support change analysis studies at a global scale.

**Fig. 3:**
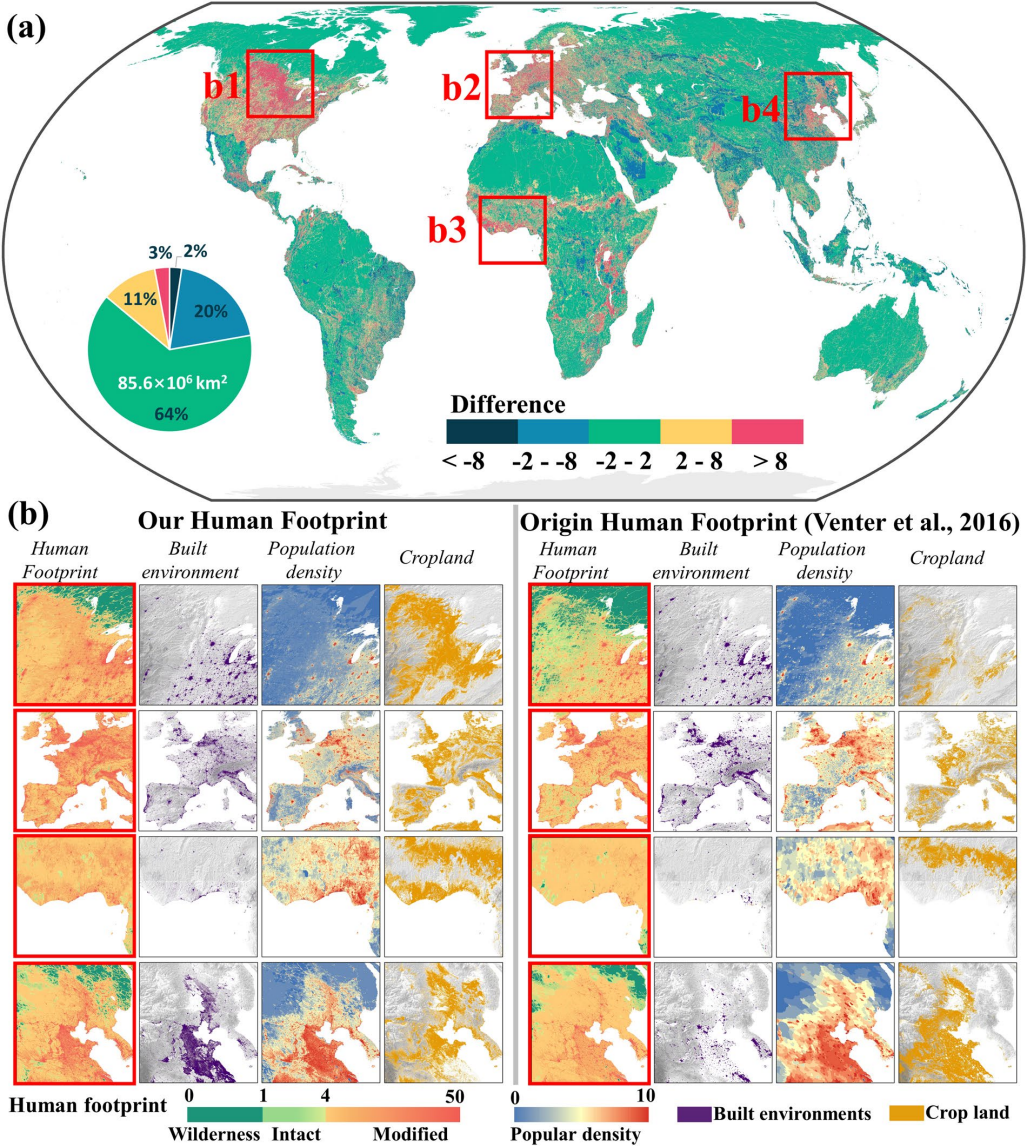
Difference of Human Footprint datasets between our result and the original map developed by Venter *et al*.^13^ in 2009 (**a**). Enlarged views in representative regions are presented in (**b**), with a spatial extent of 2000 km × 2000 km.

Compared to the original Human Footprint map, our results significantly improved those underestimated regions due to the improved quality of human pressure data from built environment, population density, and cropland (Fig. 3). The difference between the original and our Human Footprint for 2009 is mainly less than two (see green areas in Fig. 3a). We selected four representative regions for illustration in North America, Europe, Africa, and China (Fig. 3b). Compared to the 1 km NTL data used in Venter *et al*.^13^, our built environment pressure extracted from 30 m Landsat images can identify small human settlements clearly, especially in Europe and China. Besides, there is a distinct difference in the spatial pattern of cropland in North America and Africa, which is likely attributable to their inputs. For example, the cropland quality in ESA CCI is notably higher than Global Land Cover Map for 2009 (GlobCover 2009)^62^ used in Venter *et al*.^13^ regarding their spatial patterns and the temporal dynamics. In addition, the raw resolution of population density in Venter *et al*.^13^ is 4 km with two phases (i.e., 1990 and 2010), making it challenging to reflect the pressure from humans.

**Fig. 4:**
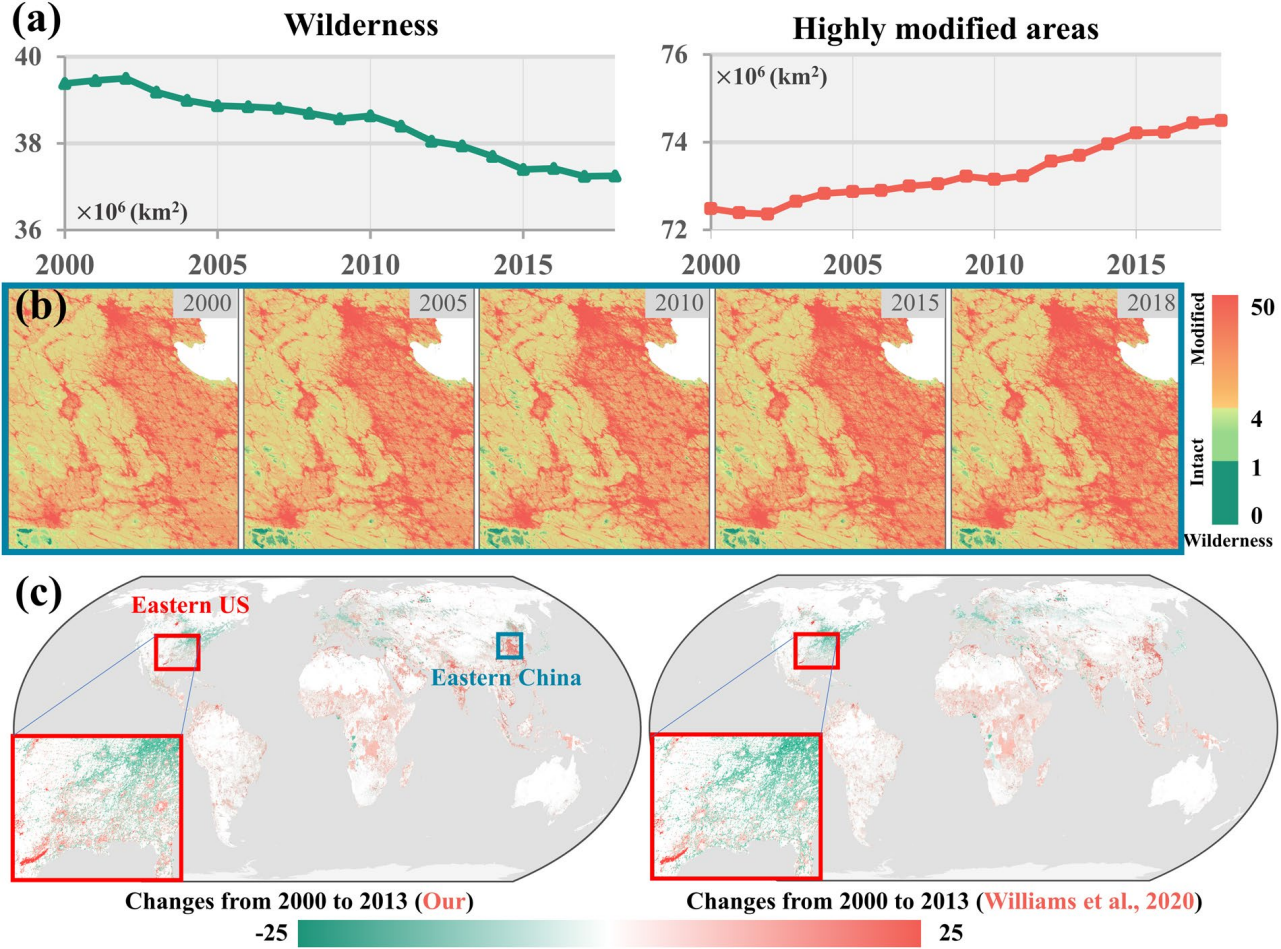
The temporal trend of global wilderness and highly modified areas from 2000 to 2018 (**a**), with detailed dynamics of Human Footprint maps in China with enlarged views (**b**) and the change of our and Williams’s^18^ Human Footprint from 2000 to 2013 (**c**). Note: the red and blue boxes in (**c**) indicate the Eastern US and Eastern China (extent in (**b**)), respectively.

Our results can reveal a continuous change of Human Footprint records (Fig. 4). From 2000 to 2018, the human pressure on 39.4% of the wilderness (i.e., without human intervention) continues to increase, of which 2.1 million km^2^ have been transformed into intact or human-modified areas (Fig. 4a). Specifically, there has been an increasing temporal trend in human-modified areas worldwide over the past decades, such as in China (Fig. 4b). Due to the global urbanization and population mitigation from rural to urban, the human pressure in rapidly developing regions is notably increased over the past decades. In addition, changes in Human Footprint (i.e., increase and decrease) from 2000 to 2013 in our derived results are consistent with Williams’s result^18^. Due to different sources of characterizing the built environment, there are some differences in detailed spatial maps from these two results (see enlarged snapshots in Fig. 4c). In general, regions with increasing human pressure are mainly distributed in Southeast Asia, Africa, and South America (Fig. 4c).

**Fig. 5:**
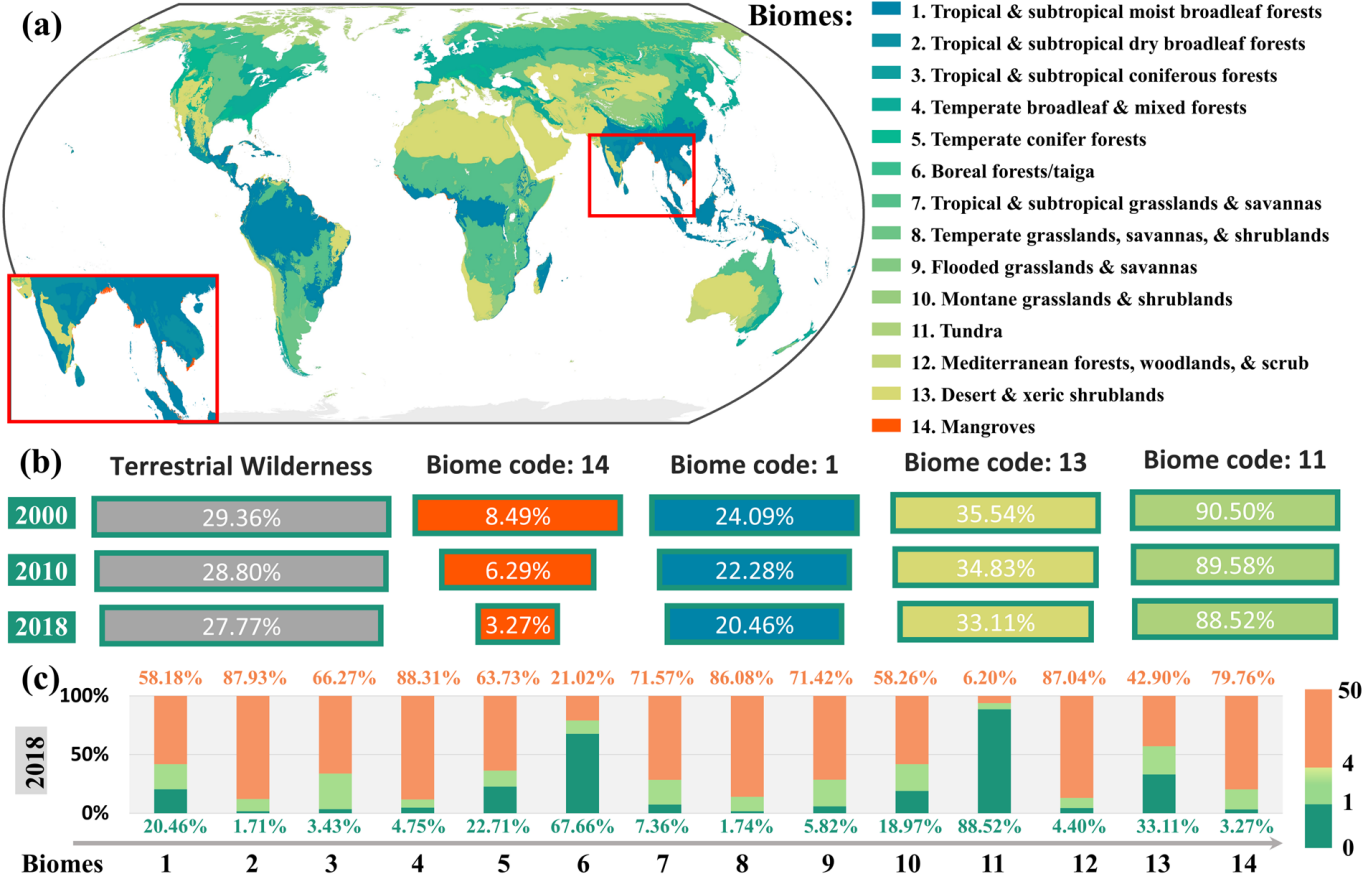
The spatial distribution of global terrestrial biomes (**a**), the changes of the wilderness of typical biomes (**b**), and the proportions of different categories (i.e., wilderness, intact, and modified) in terrestrial biomes in 2018 (**c**).

Global wilderness is declining in most terrestrial biomes^27^, especially in biomes dominated by mangroves in the coastal area of Asia (Fig. 5). This phenomenon is closely related to human activities. From 2000 to 2018, the wilderness of Mangroves (biome 14) reduced by 5.22%. Besides, other primary biomes with a noticeable decrease of wilderness are Tropical & subtropical moist broadleaf forests (biome 1), Desert & xeric shrublands (biome 13), and Tundra (biome 11), with declined proportions as 3.63%, 2.43%, and 1.98%, respectively (Fig. 5b). Globally, terrestrial biomes that suffer severe risks are Temperate grasslands, savannas, & shrublands (biome 8), and Tropical & subtropical dry broadleaf forests (biome 2), and their proportions of wilderness loss are less than 2% in 2018 (Fig. 5c). The rapid decline of wilderness challenges the realization of global environmental protection targets such as the 20 Aichi targets^63,64^.

## Usage Notes

The annual and continuous Human Footprint data are essential to monitor human pressure for studies relevant to species extinction risk^3^, conservation science^12,22^, and human development potential^65^. The updated human pressure variables, such as the GAIA, WorldPop, land cover, and global harmonized NTL datasets, enable the mapping of temporally consistent Human Footprint. Using these new variables to characterize human pressures, we developed the global annual terrestrial Human Footprint datasets from 2000 to 2018. The accuracy assessment revealed a good agreement between our Human Footprint and previous datasets at different years (i.e., 2000, 2005, 2009, 2010, 2013, and 2016). The definition used in our products is consistent with existing studies, enabling its wide applications with time series analyses in relevant studies, such as biodiversity conservation^64^, landscape planning^14^, and resources recycling^66^. Besides, the annual maps used in the scoring system can provide the temporal trend information of human pressures over the long term in the future.

## Data Availability

The programs used to generate all the results were Python (3.11) and ArcGIS (10.4). Analysis scripts are available on GitHub (https://github.com/HaoweiGis/humanFootprintMapping/).
